# Regulation of Cell Function and Myeloid‐Derived Suppressor Cell Chemotaxis by hsa_circ_0006466‐miR‐1286‐*PDGFRA*/*B* Axis in Triple Negative Breast Cancer

**DOI:** 10.1155/ancp/2285318

**Published:** 2026-02-13

**Authors:** Fengqin Gao, Mingkai Gong, Yufeng Cao, Xiaoyi Liu, Jian Cui, Bing Du, Rongrong Dou, Chuankui Wei, Hongming Song

**Affiliations:** ^1^ Breast Disease Center, The Affiliated Hospital of Qingdao University, Qingdao, Shandong, China, qdu.edu.cn; ^2^ Department of Oncology Center 3, Qingdao Hiser Hospital Affiliated of Qingdao University (Qingdao Traditional Chinese Medicine Hospital), Qingdao, Shandong, China; ^3^ Qingdao Medical College, Qingdao University, Qingdao, Shandong, China, qdu.edu.cn; ^4^ Department of General Surgery, The Second Affiliated Hospital of Shandong First Medical University, Taian, Shandong, China, natureindex.com

**Keywords:** circRNA, miR-1286, myeloid-derived suppressor cell, *PDGFRA*, *PDGFRB*, triple negative breast cancer

## Abstract

**Background:**

Circular RNAs (circRNAs) play critical regulatory roles in diverse biological processes of breast cancer. This study aimed to investigate the circRNAs potentially related to immunity in triple‐negative breast cancer (TNBC).

**Methods:**

We identified the dysregulated circRNAs in breast cancer using Gene Expression Omnibus (GEO) datasets. Then, their targeting microRNAs (miRNAs) were searched and overlapped with the miRNAs upstream of immune‐associated genes. An immune‐related circRNA‐miRNA‐mRNA network was constructed. Hsa_circ_0006466‐miR‐1286‐*PDGFRA*/*B* axis was verified from perspectives of expression levels, binding relationships, cell functions, and regulation on myeloid‐derived suppressor cells (MDSCs).

**Results:**

A total of 28 circRNAs were identified to be dysregulated and related to the immune system in TNBC. The immune‐related circRNA‐miRNA‐mRNA network consisted of 28 circRNAs, 106 miRNAs, and 205 mRNAs. Hsa_circ_0006466 and *PDGFRA*/*B* mRNA were upregulated in TNBC patients and positively correlated with MDSC levels. miR‐1286 was downregulated in TNBC patients and negatively correlated with MDSC levels. Cotransfection experiments, luciferase reporter assay, and RNA pull‐down analyses confirmed hsa_circ_0006466‐miR‐1286‐*PDGFRA*/*B* axis. Hsa_circ_0006466 inhibited TNBC cell migration/invasion and proliferation via miR‐1286‐*PDGFRA*/*B*. MDSC differentiation from THP‐1 and chemotaxis in TNBC cell medium were moderated by hsa_circ_0006466‐miR‐1286‐*PDGFRA*/*B* axis.

**Conclusions:**

Our study identifies hsa_circ_0006466 as an immune‐related circRNA in TNBC, functioning as a ceRNA to sponge miR‐1286 and upregulate *PDGFRA*/*B* expression, thereby promoting MDSC‐mediated immunosuppression and TNBC progression. Targeting this axis may offer a dual therapeutic strategy to suppress TNBC aggressiveness and reverse immune evasion.

**Summary**

• Hsa_circ_0006466‐miR‐1286‐PDGFRA/B axis was dysregulated in triple‐negative breast cancer (TNBC).
• Hsa_circ_0006466‐miR‐1286‐PDGFRA/B axis was related to immune in TNBC.
• Hsa_circ_0006466‐miR‐1286‐PDGFRA/B axis mediated TNBC progression.
• Hsa_circ_0006466‐miR‐1286‐PDGFRA/B axis mediated MDSC differentiation and chemotaxis.

## 1. Introduction

Female breast cancer emerges as one of the cancer types most frequently diagnosed among adults in most high‐income countries [1]. Accounting for about 12% of all female cancer cases, breast cancer has overtaken lung cancer as the most common cancer among women globally [2]. Female breast cancer deaths are a major cancer killer [3, 4]. Triple‐negative breast cancer (TNBC), characterized by the absence of estrogen receptor (ER), progesterone receptor (PR), and HER2 amplification, remains the most aggressive and therapeutically challenging breast cancer subtype [5]. Chemotherapy remains the mainstay for TNBC due to the lack of hormone/HER2 targets [6]. However, resistance and toxicity are common issues. Despite advances in chemotherapy and immunotherapy, TNBC exhibits a high propensity for early metastasis, immune evasion, and poor clinical outcomes, partly driven by the immunosuppressive tumor microenvironment (TME) [6, 7]. Myeloid‐derived suppressor cells (MDSCs), key orchestrators of immune suppression in TNBC, promote tumor progression by inhibiting cytotoxic T‐cell activity and fostering angiogenesis [7–9]. However, the molecular mechanisms regulating MDSC recruitment in TNBC are incompletely understood, limiting the development of targeted immunomodulatory strategies.

Noncoding RNAs, particularly circular RNAs (circRNAs), have emerged as critical regulators of tumorigenesis and immune evasion [10–12]. By acting as competitive endogenous RNAs (ceRNAs), circRNAs sequester microRNAs (miRNAs), thereby modulating the expression of downstream mRNAs involved in oncogenic signaling [13, 14]. Recent studies implicate circRNA‐miRNA‐mRNA networks in TNBC progression, including metastasis and chemoresistance [15, 16]. For instance, circTADA2A promotes TNBC stemness by sponging miR‐203a‐3p to upregulate SOCS3 [17], while circANKS1B enhances invasion via the miR‐148a‐3p/*USF1* axis [18]. Despite these advances, the role of circRNA‐driven networks in shaping the TNBC immune landscape, particularly MDSC dynamics, remains unexplored. Recent advancements in RNA sequencing technologies and multiomics integration have revolutionized the identification of noncoding RNA networks in cancer biology [18]. In particular, multipartite regulatory networks, encompassing circRNAs, miRNAs, and mRNAs, have unveiled novel mechanisms by which circRNAs drive oncogenesis through competitive RNA–RNA interactions [18]. However, systematic analyses of such networks in TNBC remain limited.

In this study, we leveraged publicly available breast cancer transcriptomic datasets (GEO) to comprehensively profile aberrantly expressed circRNAs and miRNAs in TNBC. Immune‐related genes were retrieved and traced back for potential immune‐associated miRNAs and circRNAs, to construct an immune‐related circRNA‐miRNA‐mRNA network in TNBC. Then, our focus centered on hsa_circ_0006466‐miR‐1286‐*PDGFRA*/*B* axis from perspectives of expression levels, binding relationships, cell functions, and regulation on MDSCs.

## 2. Materials and Methods

### 2.1. Data Acquisition and Processing

Transcriptomic datasets for TNBC were retrieved from the GEO database (https://www.ncbi.nlm.nih.gov/geo/). Specifically, circRNA expression profiles were obtained from the GSE182471 and GSE229572 datasets, while TNBC‐associated miRNAs were identified using GSE248460. Differential expression analysis was performed using the GEO2R online tool and the limma R package, with significance thresholds set at |log2(fold change)| > 1 and adjusted *p* < 0.05.

To refine TNBC‐specific molecular signatures, candidate genes were curated from the GeneCards database (https://www.genecards.org/), retaining those with an average Relevance score ≥5. Immune‐related genes were further annotated using the ImmPort portal (https://www.immport.org/), a publicly accessible repository for immunology datasets.

### 2.2. Screening of cicRNA‐Targeting miRNAs and Downstream Genes

1. The possible targeting miRNAs for differentially expressed cicRNAs in TNBC were predicted using the circbank database. The intersection of circRNA‐targeting miRNAs with differentially expressed in TNBC was obtained, prepared for constructing the circRNAs/miRNAs network.
2. The target genes that may be regulated by miRNAs were predicted using miRWalk version 2, and then the intersection was taken with TNBC‐ and immune‐related genes, prepared for constructing lncRNAs/miRNA/gene network.

### 2.3. Construction of Regulatory Network

CircRNAs/miRNA/gene network was constructed using Cytoscape software.

### 2.4. Patient Enrollment and Sample Collection

Our study protocols were approved by the Institutional Ethics Committees of the Hospital (No. QYFY‐WZLL‐30696). All participants provided informed consent prior to inclusion in the study. Fifty patients diagnosed with TNBC (mean age 47.8 ± 7.6) were enrolled from the Breast Disease Center of the Affiliated Hospital of Qingdao University (Qingdao, China) between July 2020 and July 2022. Peripheral blood samples were collected from all participants. The inclusion criteria were as follows: diagnosis consistent with TNBC diagnostic standards; histopathological confirmation of TNBC; availability of complete clinicopathological records; and comprehensive laboratory test results. The exclusion criteria were as follows: (1) history of other malignant tumors; (2) presence of severe concurrent medical conditions (including heart, liver, or renal failure); (3) incomplete clinical or follow‐up data; (4) pregnancy or lactation; and (5) receipt of any anti‐cancer therapy prior to enrollment. Additionally, 50 age‐matched (mean age 46.3 ± 5.4 years) healthy individuals who underwent routine health check‐ups at the same institution during the aforementioned period were recruited as the control group. Clinical data and MDSC profiles were systematically collected from TNBC patients for subsequent analysis.

### 2.5. Cell Culture

The TNBC cell lines (HCC1937 and MDA‐MB‐231) were utilized in this study. HCC1937 cells and MDA‐MB‐231 cells were obtained from the Chinese Academy of Sciences Cell Bank (Shanghai, China), where they were characterized based on mycoplasma detection and STR (short tandem repeat) amplification. HCC1937 and MDA‐MB‐231 cell lines were cultured in DMEM (Gibco, USA) containing 10% FBS and 1% PS. All cell lines were cultured in a 5% CO_2_ incubator at 37°C. All cell lines were periodically tested to be mycoplasma‐free, and maintained at a low passage number (<10 passages).

### 2.6. Cell Transfection

Small interfering RNAs (siRNAs) targeting hsa_circ_0006466, *PDGFRA*, and *PDGFRB*, along with non‐targeting scrambled siRNA controls, were designed and synthesized by GenePharma Co., Ltd. (Shanghai, China). Similarly, miR‐1286 mimics, inhibitors, and their respective negative controls (mimic‐NC; anti‐NC) were procured from GenePharma.

For transfection, HCC1937 and MDA‐MB‐231 cells were seeded into 6‐well plates and grown to 70%–80% confluence. Transient transfection was performed using Lipofectamine 3000 reagent (Invitrogen, Carlsbad, CA, USA) using siRNAs, miRNA mimics/inhibitors, or the negative controls at a final concentration of 50 nM according to the manufacturer’s protocol.

### 2.7. Luciferase Reporter Assays

To validate direct interactions between miR‐1286 and hsa_circ_0006466 or *PDGFRA*/*PDGFRB*, wild‐type (wt) and mutated (mut) sequences containing predicted miR‐1286 binding sites were cloned into the pmirGLO dual‐luciferase vector (Promega, Madison, WI, USA). All constructs were verified by sequencing. For luciferase assays, HCC1937 and MDA‐MB‐231 cells were seeded in 24‐well plates (5 × 10^4^ cells/well) and co‐transfected in triplicate with: 100 ng of luciferase reporter plasmid (wt or mut hsa_circ_0006466/*PDGFRA*/*PDGFRB*); 100 nM miR‐1286 mimic or negative control miRNA; 10 ng Luciferase vectors (Promega; USA). Transfections were performed using Lipofectamine 3000 (Invitrogen, USA) according to the manufacturer’s protocol. At 48 h post‐transfection, luciferase activities were quantified using the Dual‐Luciferase Reporter Assay System (Promega) on a GloMax Navigator microplate luminometer (Promega). Firefly luciferase signals were normalized to Renilla values for each sample.

### 2.8. RNA Pull‐Down Assay

To confirm direct binding between miR‐1286 and hsa_circ_0006466 or *PDGFRB*, a biotinylated antisense oligonucleotide (ASO)‐based RNA pull‐down assay was performed. Three nonoverlapping biotinylated ASOs targeting the backsplice junction region of hsa_circ_0006466 (to ensure circRNA specificity) and the 3^′^UTR of *PDGFRB* (NM_002609.4) were designed using OligoWalk (RNAstructure 6.4). A scrambled biotinylated probe served as the negative control. Briefly, HCC1937 and MDA‐MB‐231 cells were lysed in NP‐40 buffer supplemented with RNase inhibitor (Takara). Lysates (500 µg protein/sample) were precleared with streptavidin magnetic beads (Invitrogen, Carlsbad, USA) at 4°C for 2 h to reduce nonspecific binding. Subsequently, 1 µg of biotinylated ASO probes (RiboBio, Guangzhou, China) was added to the lysates and incubated overnight at 4°C with gentle rotation. RNA‐protein complexes were captured by streptavidin beads, washed five times with high‐salt buffer (300 mM NaCl), and eluted in TRIzol LS reagent (Invitrogen). Enriched RNAs were detected by RT‐qPCR.

### 2.9. Cell Proliferation Assay

Cell proliferation was assessed using the Cell Counting Kit‐8 (CCK‐8; Biovision, Milpitas, CA, USA; Cat# K1018). Briefly, HCC1937 and MDA‐MB‐231 cells transfected with siRNA targeting hsa_circ_0006466, miR‐1286 mimic/inhibitor, or respective controls were seeded into 96‐well plates (2 × 10^4^ cells/well) in quadruple. After incubation for 0, 24, 48, and 72 h under standard culture conditions (37°C, 5% CO_2_ for HCC1937 and MDA‐MB‐231), 10 μL of CCK‐8 reagent was added to each well, for a further incubation of 2 h. Absorbance was measured at 450 nm. A SpectraMax M5 microplate reader was used.

### 2.10. Cell Migration and Invasion Assay

Cell migratory and invasive capacities were evaluated using 24‐well Transwell chambers (8 μm pore size; Corning, NY, USA; Cat# 3422) coated with or without Matrigel (BD Biosciences, San Jose, CA, USA; Cat# 356234) for invasion and migration assays, respectively. Transfected HCC1937 and MDA‐MB‐231 cells were serum‐starved for 12 h. With cells in serum‐free medium loaded in the upper chamber, 600 μL complete medium containing 10% FBS was transferred into the lower chamber. Cells were allowed to migrate/invade for 12 h (migration) or 16 h (invasion) under standard culture conditions. Non‐migrated cells were removed. Migrated/invaded cells were fixed. After staining, cells were imaged under an inverted microscope (Nikon Eclipse Ti). Cells in five random fields per insert were counted using ImageJ 1.53k (NIH, USA). Data were normalized to control groups.

### 2.11. RT‐qPCR Analysis

Total RNA was isolated from TNBC cells or clinical samples using the E.Z.N.A. Total RNA Kit (Omega Bio‐Tek, USA). RNA concentration and purity were determined spectrophotometrically (NanoDrop 2000; Thermo Fisher Scientific, USA), with A260/A280 ratios between 1.8 and 2.1 considered acceptable. First‐strand cDNA synthesis was performed using 1 µg of total RNA with the High‐Capacity cDNA Reverse Transcription Kit (Thermo Fisher Scientific). qPCR amplification was carried out in triplicate using SYBR Green Master Mix (Thermo Fisher Scientific) on a StepOne Plus Real‐Time PCR System (Applied Biosystems, Foster City, CA, USA). Relative expression levels were calculated using the 2^−ΔΔCt^ method. GAPDH was used as an internal loading control to normalize the PCR products of circRNA and mRNAs, while U6 was used as an internal loading control to normalize the PCR products of miRNAs. Results represent four independent experiments and are expressed as fold changes relative to control groups.

### 2.12. MDSC‐Like Differentiation From THP‐1

Conditioned medium (CM) was collected from TNBC cells transfected with hsa_circ_0006466 siRNA, while control medium was from cells transfected with siRNA negative control. With or without CM from TNBC cells, THP‐1 cells were incubated with 100 ng/mL of Granulocyte colony‐stimulating factor and 20 ng/mL of IL‐4 (Sigma–Aldrich, USA). Cells were seeded in Nunc 12‐well plates (Thermo Fisher Scientific, USA) at 5 × 10^5^ cells/well in 1 mL RPMI‐1640 supplemented with 100 ng/mL recombinant human G‐CSF (PeproTech, Rocky Hill, USA), 20 ng/mL IL‐4 (Sigma–Aldrich, USA), 30% (v/v) TNBC cell‐derived CM, and 10% FBS. Cells were maintained for 7 days at 37°C with 5% CO_2_. On day 3, differentiated cells were split into fresh wells at a 1:2 ratio to maintain optimal density (2.5 × 10^5^ cells/well in 1 mL fresh medium). Control groups received cytokine‐free medium with 30% RPMI‐1640 instead of CM. After stimulation to differentiate into THP1‐MDSC‐like cells, the cells were harvested and labeled with antibodies anti‐CD11b (BD Biosciences, USA), CD33 (BD Biosciences, USA), CD16 (BD Biosciences, USA), and Lin1 (BD Biosciences, USA). Detection was taken in the BD LSRFortessa X‐20 cell analyzer Cytometer equipment (Becton, Dickinson and Company, USA). Cells CD11b^+^/CD33^+^/CD16^-^/Lin1^-^ were gated as the differentiated population of THP1‐MDSC‐like cells, while cells negative for these surface markers were gated as the undifferentiated population. Three biological replicates were performed.

### 2.13. MDSC Isolation and Chemotaxis Assay

Human MDSCs (PMN‐MDSCs and M‐MDSCs) were isolated from the peripheral blood of TNBC patients using a MDSC Isolation Kit (Miltenyi Biotec). 3.0 × 10^5^ of each set of MDSC was loaded in the upper chamber of a CytoSelect 24‐well Cell Migration Assay plate (Cell Biolabs). Medium from TNBC cells transfected by hsa_circ_0006466 siRNA (CM) or siRNA negative control (lower) was collected and cocultured with MDSC (upper). Following a duration of 6 h, a dissociation of the migratory cells was conducted utilizing Cell Detachment Buffer. Thereafter, a quantifiable procedure was initiated, employing a CyQuant GR Fluorescent Dye Solution for quantitative analysis.

### 2.14. Statistical Analysis

All statistical analyses were performed using GraphPad Prism 9.0 (GraphPad Software, San Diego, CA, USA). Data from three independent experiments are presented as mean ± standard deviation (SD). The threshold for statistical significance was set at *p* < 0.05, with multiple testing corrections applied where appropriate. Specific analytical approaches included: two‐group comparisons: Unpaired Student’s *t*‐test (parametric) or Mann–Whitney *U* test (nonparametric), depending on normal distribution assessed by Shapiro–Wilk test; multigroup comparisons: one‐way ANOVA with Tukey’s post hoc test or two‐way ANOVA with Bonferroni correction. Correlation studies: Spearman’s rank (nonparametric) coefficients. Significance was indicated unless >0.05, with significance denoted as  ^∗^ *p* < 0.05,  ^∗∗^ *p* < 0.01, and  ^∗∗∗^ *p* < 0.001.

## 3. Results

### 3.1. Identification of Differentially Expressed circRNAs in Breast Cancer

**Figure 1:**
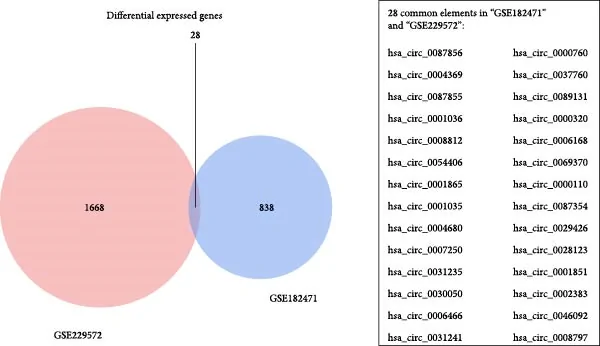
The intersected dysregulated circRNAs between the GSE229572 and the GSE182471 datasets.

Through integrated analysis of circRNA expression profiles from two independent breast cancer datasets (GSE229572 and GSE182471), we systematically identified dysregulated circRNAs using predefined screening criteria (|log2(fold change)| > 1; *p* < 0.05). Comparative analysis revealed that GSE229572 resulted in 1668 differentially expressed circRNAs (892 upregulated and 776 downregulated), while GSE182471 resulted in 838 DECs (467 upregulated, 371 downregulated). Venn diagram analysis identified 28 consensus circRNAs consistently dysregulated across both datasets (Figure 1).

### 3.2. The TNBC‐ and Immune‐Related circRNA/miRNA/mRNA Network

**Figure 2:**
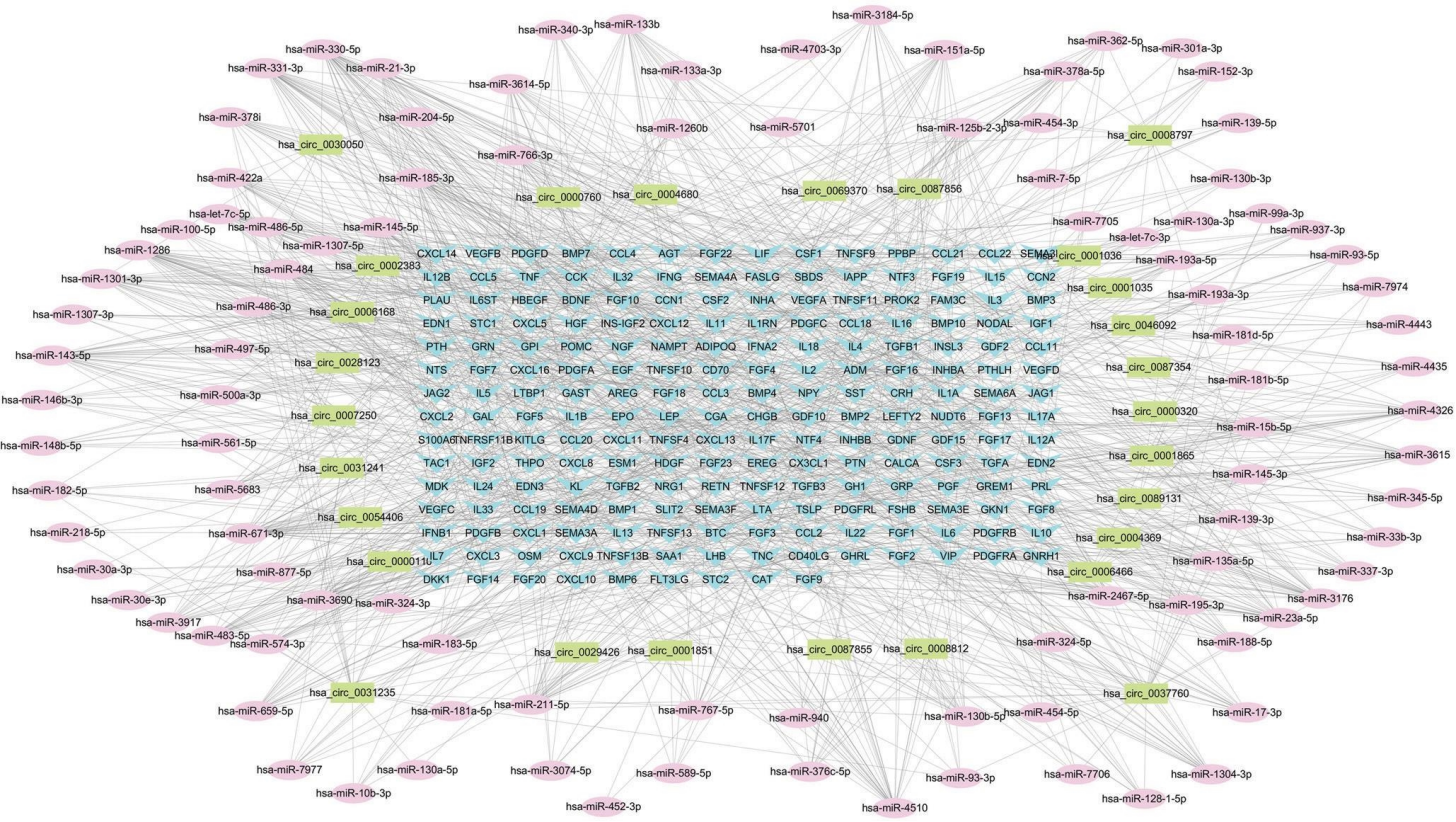
A immune‐related circRNA‐miRNA‐mRNA regulatory network in TNBC. The network consisting of 28 circRNAs, 106 miRNAs, and 205 mRNAs was generated by Cytoscape 3.9.0.

Then, we take the intersection of differentially expressed miRNAs in TNBS (GSE248460), circRNA‐target miRNAs, and immune‐related miRNAs, and a total of 106 miRNAs were obtained (Figure S1). These 106 miRNAs linked 28 of upstream circRNAs and 205 of downstream mRNAs that related to immune (Figure 2). The constructed network contained 338 nodes and 1305 edges, with an average number of neighbors of 7.768 (Figure 2).

### 3.3. Dysregulation of the hsa_circ_0006466‐miR‐1286‐PDGFRA/B Axis in TNBC

We systematically profiled the expression of hsa_circ_0006466, miR‐1286, and *PDGFRA*/*B* across 50 TNBC clinical specimens and matched normal tissues. We found that hsa_circ_0006466 expression was significantly elevated in TNBC tissues compared to adjacent normal controls (*p* < 0.001; Figure 3A), using RT‐qPCR. MiR‐1286 levels were markedly reduced in TNBC (*p* < 0.01; Figure 3B), suggesting potential tumor‐suppressive activity. Both *PDGFRA* (*p* < 0.001) and *PDGFRB* (*p* < 0.001) mRNA were upregulated in TNBC (Figure 3C and D), consistent with their roles in stromal crosstalk [19]. Then, we attempted to explore the correlation between these factors and MDSC infiltration. Hsa_circ_0006466 levels were correlated with MDSC abundance (*r* = 0.6360, *p* < 0.001; Figure 3E). miR‐1286 expression inversely associated with MDSC infiltration (*r* = −0.5860, *p* < 0.001; Figure 3F). *PDGFRA* (*r* = 0.7241) and *PDGFRB* (*r* = 0.7928) showed significant positive associations with MDSC abundance (*p* < 0.001; Figure 3G, H).

Figure 3Dysregulation of the hsa_circ_0006466‐miR‐1286‐*PDGFRA*/*B* axis in TNBC. The expression levels of hsa_circ_0006466 (A), miR‐1286 (B), *PDGFRA* mRNA (C), and *PDGFRB* mRNA (D) in TNBC patients and controls were determined using RT‐qPCR. The correlations of MDSC level with hsa_circ_0006466 (E), miR‐1286 (F), *PDGFRA* mRNA (G), and *PDGFRB* mRNA (H) in TNBC patients were analyzed using Spearman’s rank (non‐parametric) coefficients. ( ^∗∗^ *p* < 0.01,  ^∗∗∗^ *p* < 0.001.).

**Figure:**
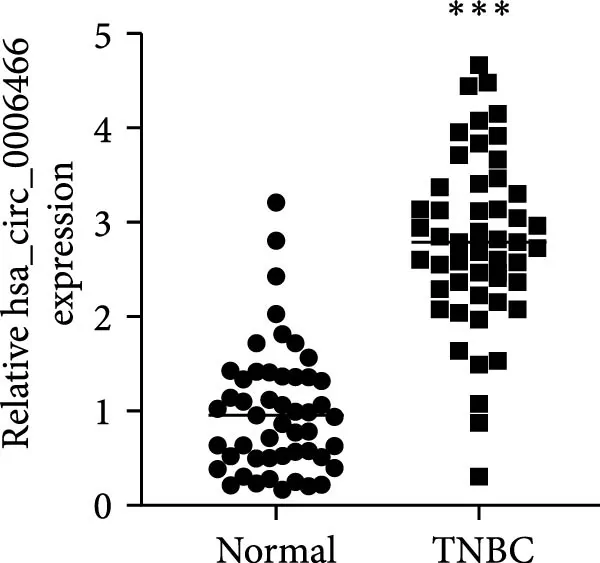
(A)

**Figure:**
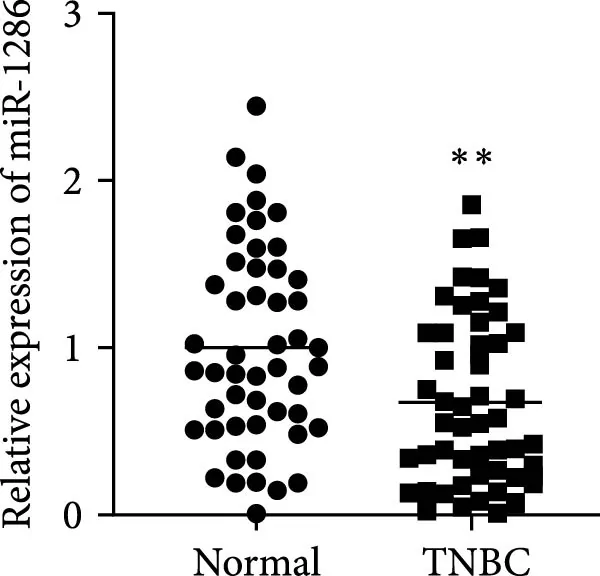
(B)

**Figure:**
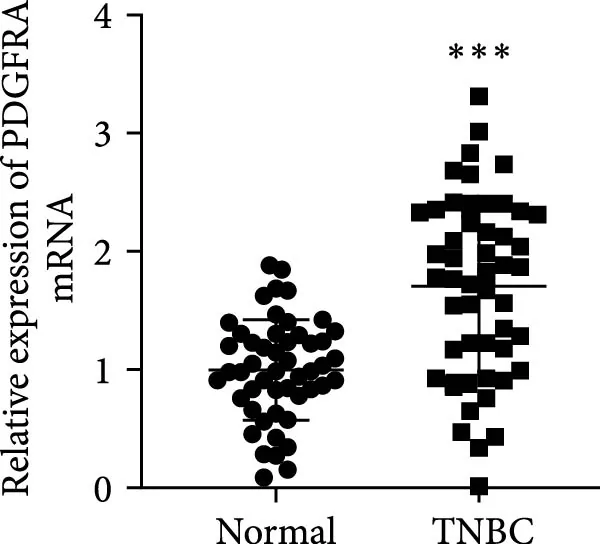
(C)

**Figure:**
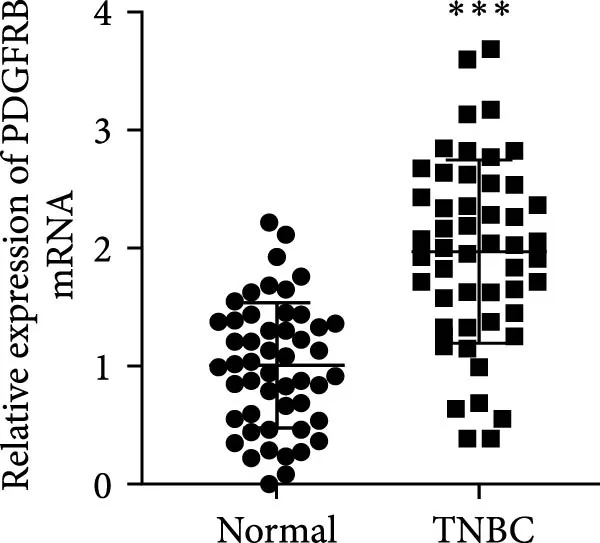
(D)

**Figure:**
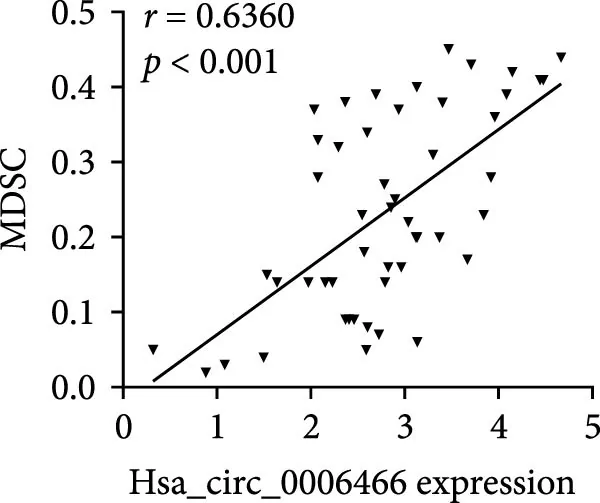
(E)

**Figure:**
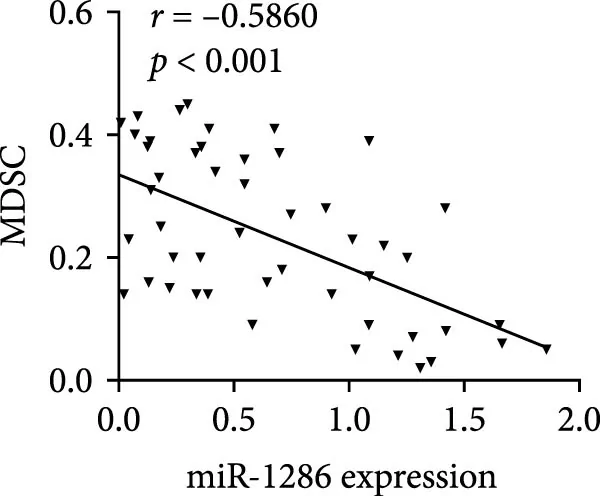
(F)

**Figure:**
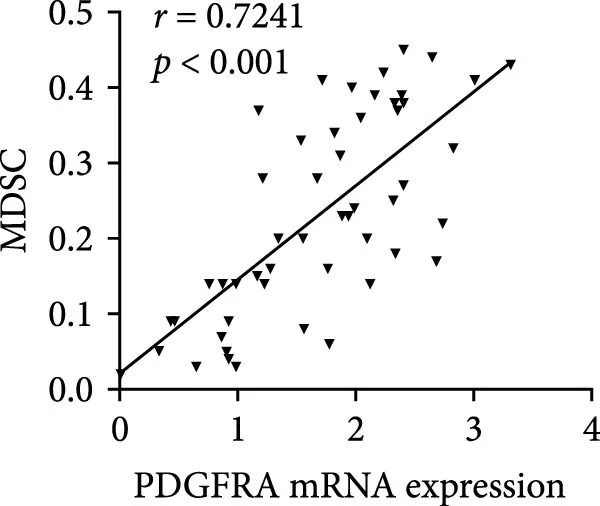
(G)

**Figure:**
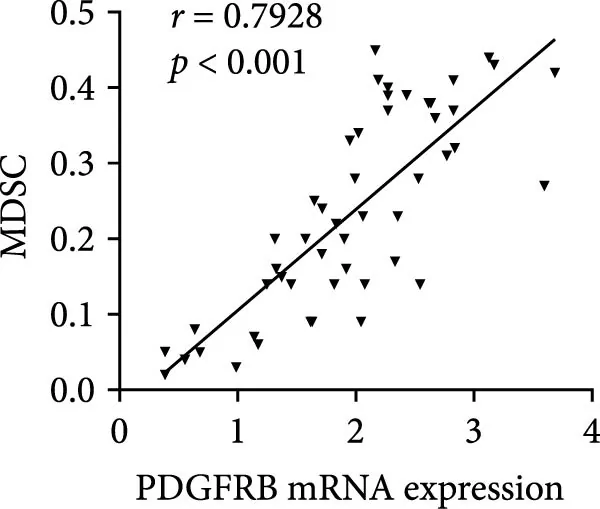
(H)

### 3.4. Validation of hsa_circ_0006466/miR‐1286/PDGFRA/B Axis

The binding sites between hsa_circ_0006466 and miR‐1286 were listed in Figure 4A. Activity measurements of luciferase after transfection revealed that luciferase was denatured after miR‐1286 overexpression (shrunk) or inhibition (aggregated) in cells carrying wild‐type hsa_circ_0006466; notably, the cells carrying mutant hsa_circ_0006466 weren’t influenced (*p* < 0.001; Figure 4B and C). To confirm direct interaction between hsa_circ_0006466 and miR‐1286, an in vitro binding assay using a biotinylated hsa_circ_0006466 in pull‐down experiments showed enrichment of miR‐1286 (*p* < 0.001; Figure 4D). Transfection with siRNA for hsa_circ_0006466 caused an increase in miR‐1286 expression (*p* < 0.01; Figure 4E). The binding sites between *PDGFRA*/*B* and miR‐1286 were listed in Figure 4F. In contrast to wild‐type *PDGFRA*, luciferase activity of mutant hsa_circ_0006466 was significantly inhibited by miR‐1286 mimics, while it was aggregated by miR‐1286 inhibitor (*p* < 0.001; Figure 4G). miR‐1286 was enriched in *PDGFRA* probe (*p* < 0.001; Figure 4H). Transfection with miR‐1286 inhibitor caused increases in *PDGFRA*/*B* expression, while miR‐1286 mimics led to reductions in *PDGFRA*/*B* expression (*p* < 0.001; Figure 4I and J).

Figure 4Hsa_circ_0006466 functioned as a molecular sponge of miR‐1286 to promote *PDGFRA*/*B*. (A) The binding sites between hsa_circ_0006466 and miR‐1286. Luciferase reporter assays of wild‐type or mutant hsa_circ_0006466 after miR‐1286 overexpression or inhibition in HCC1937 (B) and MDA‐MB‐231 (C) cells. (D) RT‐qPCR of miR‐1286 in RNA pulldown assays in TNBC cells with biotin‐labeled hsa_circ_0006466. (E) RT‐qPCR of miR‐1286 in TNBC cells transfected by hsa_circ_0006466 siRNA. (F) The binding sites between miR‐1286 and *PDGFRA*/*B*. (G) Luciferase reporter assays of wild‐type or mutant *PDGFRA* after miR‐1286 overexpression or inhibition in HCC1937 cells. (H) RT‐qPCR of miR‐1286 in RNA pulldown assays in MDA‐MB‐231 cells with biotin‐labeled *PDGFRB*. (I, J) RT‐qPCR of miR‐1286 in TNBC cells transfected by miR‐1286 inhibitor and/or hsa_circ_0006466 siRNA. ( ^∗∗^ *p* < 0.01,  ^∗∗∗^ *p* < 0.001.).

**Figure:**
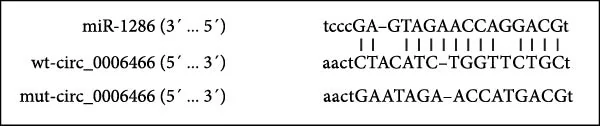
(A)

**Figure:**
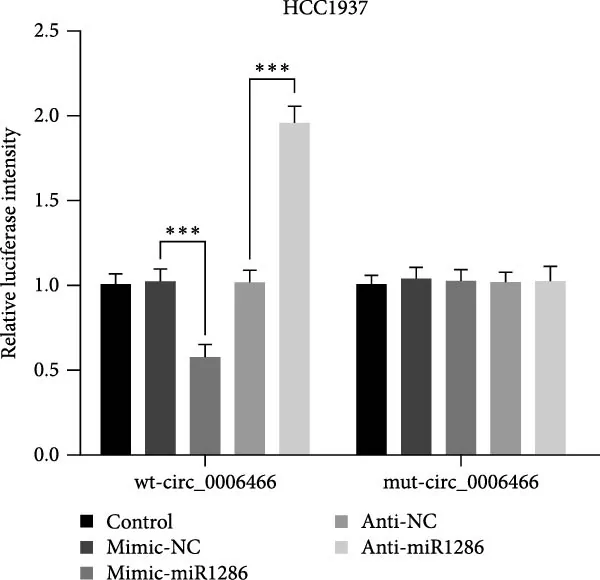
(B)

**Figure:**
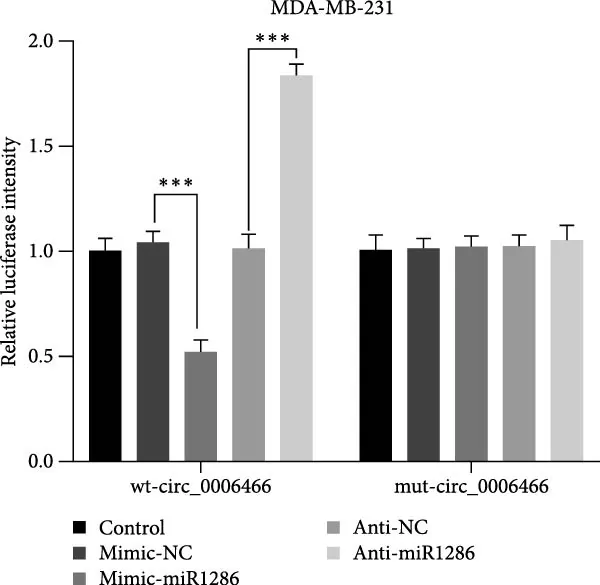
(C)

**Figure:**
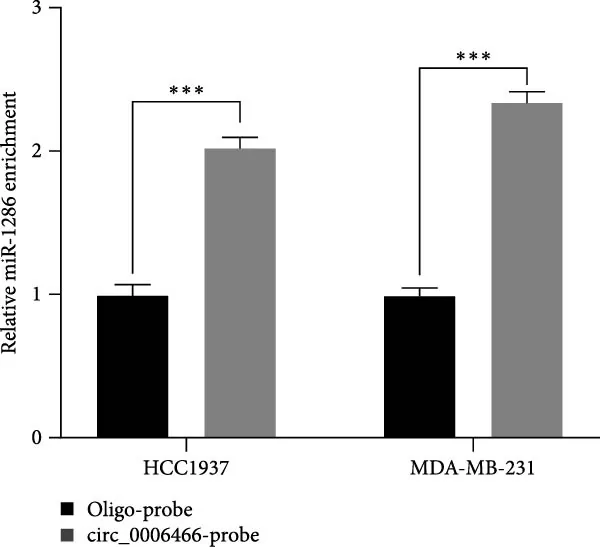
(D)

**Figure:**
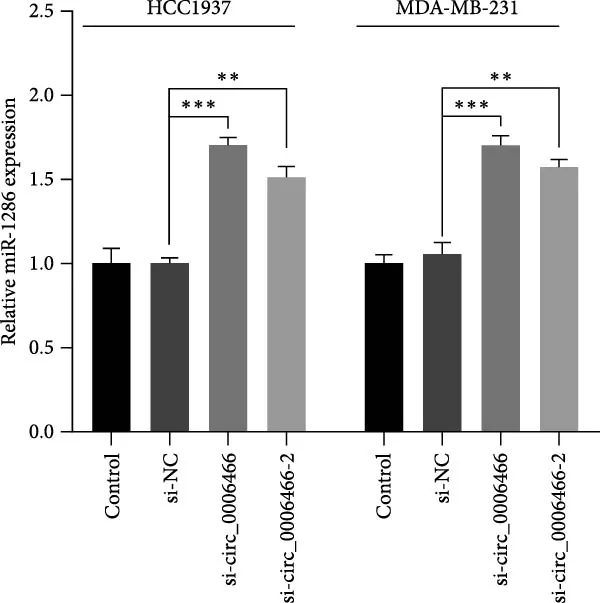
(E)

**Figure:**
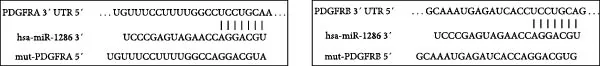
(F)

**Figure:**
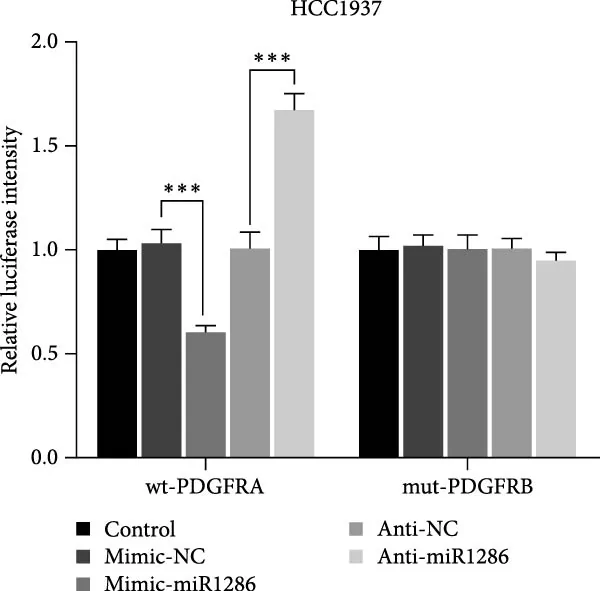
(G)

**Figure:**
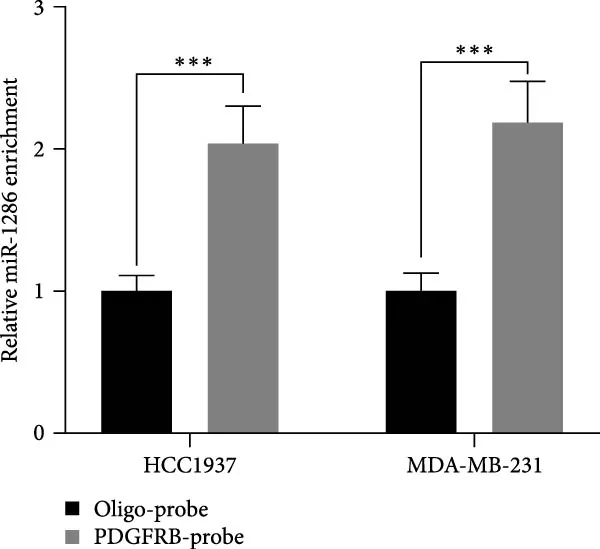
(H)

**Figure:**
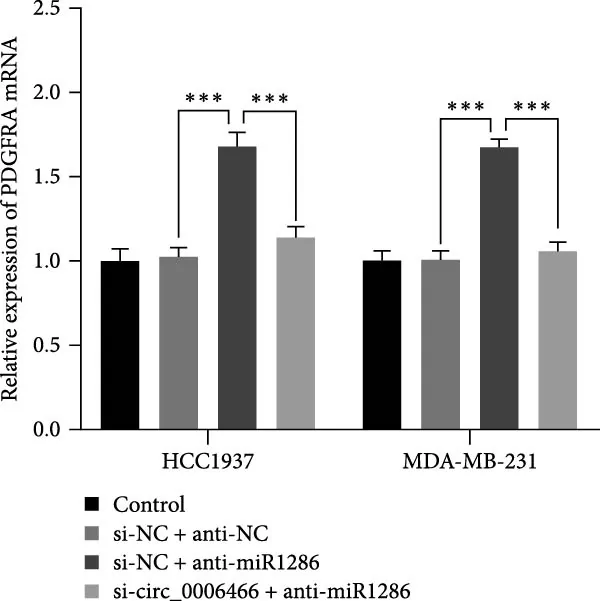
(I)

**Figure:**
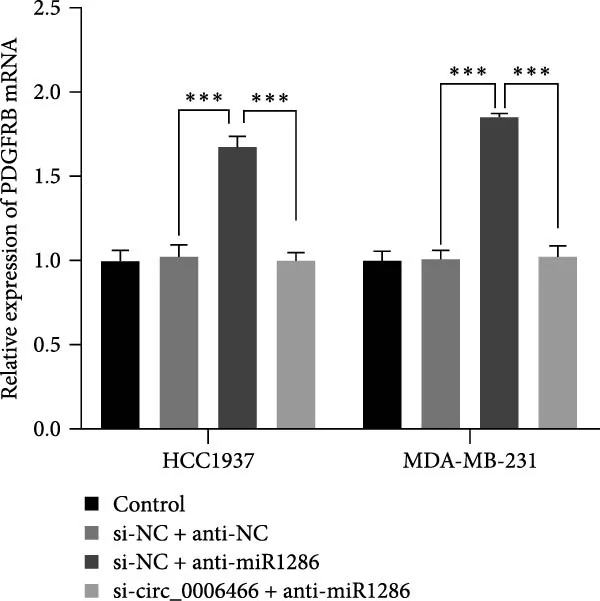
(J)

### 3.5. The Changes in the TNBC Cell Function After hsa_circ_0006466/miR‐1286/PDGFRA/B Axis Alteration

To further investigate the functional interaction between hsa_circ_0006466 and miR‐1286, rescue experiments were conducted by co‐transfection. Transfection with hsa_circ_0006466 or *PDGFRA*/*B* siRNA caused a reduction in *PDGFRA*/*B* expression, while miR‐1286 inhibitors led to aggregation in *PDGFRA*/*B* expression (*p* < 0.001; Figure 5A and B). After hsa_circ_0006466 silencing in TNBC cells, cell migration and invasion were inhibited (*p* < 0.001; Figure 5C and D). As expected, miR‐1286 inhibitor partially attenuated the inhibition of cell migration and invasion, which were inhibited again by *PDGFRA*/*B* siRNA (*p* < 0.001; Figure 5C and D). In both cells, the proliferation was decreased after hsa_circ_0006466 downregulation, but increased again after both downregulation of hsa_circ_0006466/miR‐1286 (*p* < 0.01; Figure 5E and F). Additionally, *PDGFRA*/*B* siRNA reduced the increase in cell proliferation (*p* < 0.01; Figure 5E and F).

Figure 5Cell function of hsa_circ_0006466‐miR‐1286‐*PDGFRA*/*B* axis in TNBC. (A, B) RT‐qPCR of TNBC cells for *PDGFRA*/*B* expression levels after transfection. (C) Transwell migration assays of TNBC cells after transfection. (D) Transwell invasion assays of TNBC cells after transfection. (E, F) Cell CCK‐8 proliferation assays of with TNBC cells after transfection. ( ^∗∗^ *p* < 0.01,  ^∗∗∗^ *p* < 0.001.).

**Figure:**
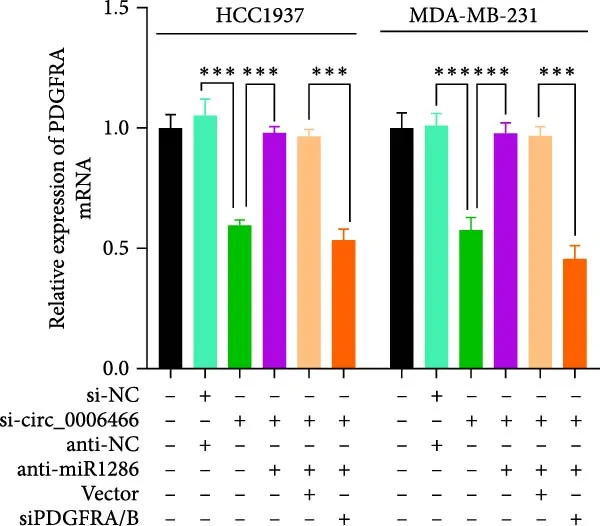
(A)

**Figure:**
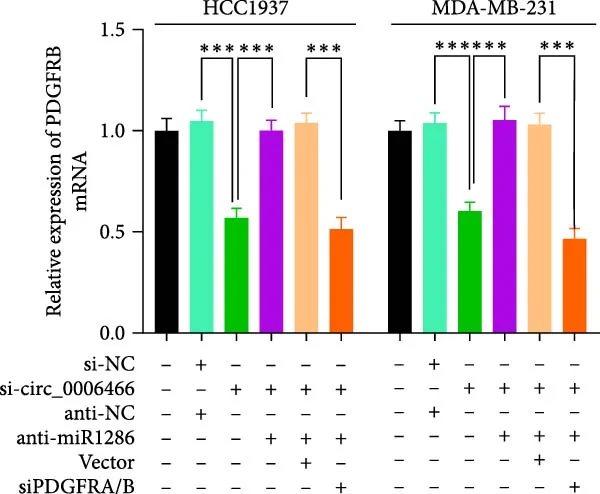
(B)

**Figure:**
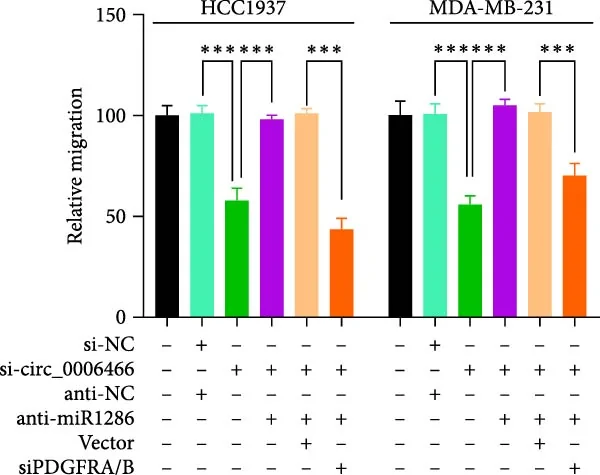
(C)

**Figure:**
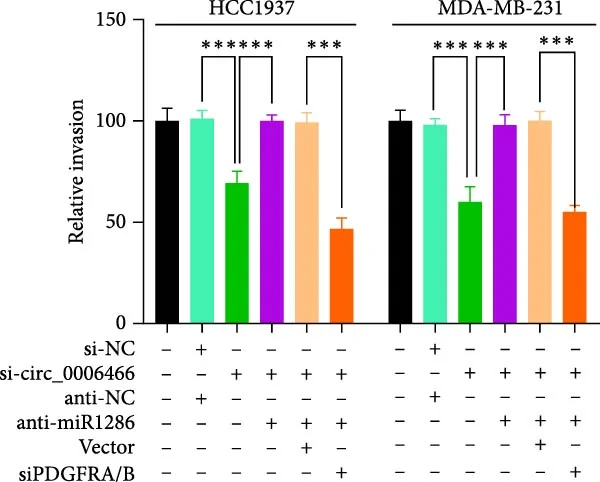
(D)

**Figure:**
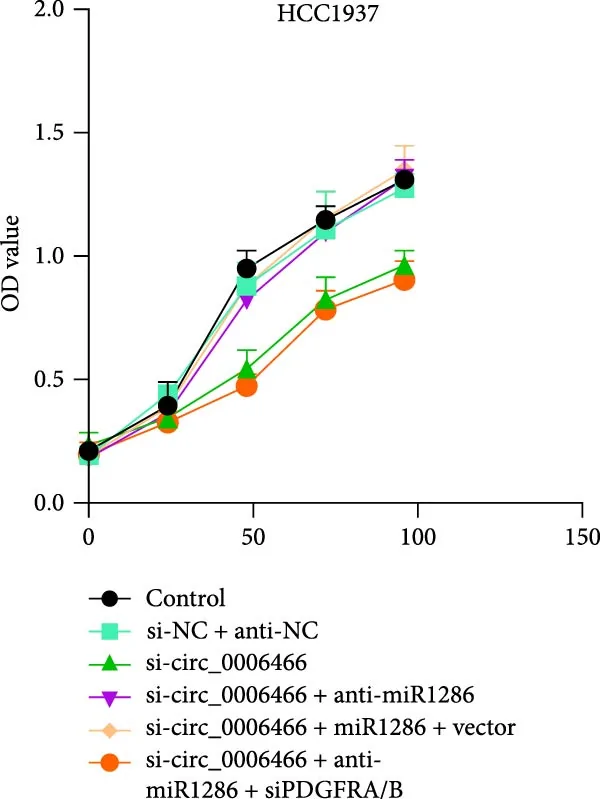
(E)

**Figure:**
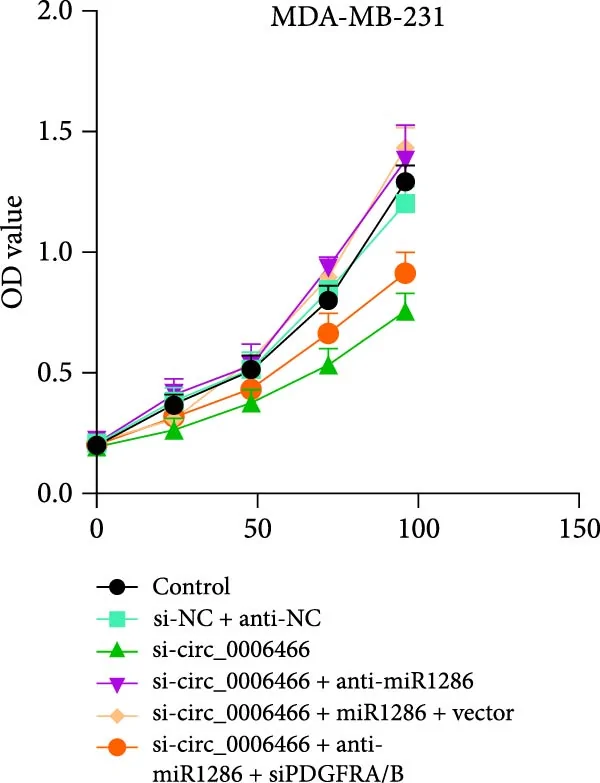
(F)

### 3.6. The Changes in MDSC Chemotaxis After hsa_circ_0006466/miR‐1286/PDGFRA/B Axis Alteration

Based on the results from the analysis of human TNBC cell culture, expression levels of CXCL1 and CXCL3 mRNAs were found to be upregulated in CM groups, when compared with those in control medium groups (*p* < 0.001; Figure 6A and B). After submitting cells to the differentiation by culture in control medium or CM groups, the number of differentiated PMN‐MDSCs was higher in CM groups than that in control medium groups (*p* < 0.01; Figure 6C and D). Then, MDSCs isolated from TNBC patients were subjected to a chemotaxis assay using CM from TNBC cells treated with hsa_circ_0006466 siRNA or control medium treated with siRNA negative control. Consequently, the number of invaded PMN‐MDSCs through the membrane was increased in the TNBC CM groups (*p* < 0.01; Figure 6E and F).

Figure 6Regulation of MDSCs by hsa_circ_0006466‐miR‐1286‐*PDGFRA*/*B* axis in TNBC. (A, B) The mRNA expression of CXCL1 and CXCL3 in medium from TNBC cells with or without transfection of hsa_circ_0006466 siRNA. (C, D) Abundance of PMN‐MDSCs and M‐MDSCs differentiated from THP‐1 under medium from TNBC cells with or without transfection of hsa_circ_0006466 siRNA. (E, F) Chemotaxis of PMN‐MDSCs and M‐MDSCs using medium from TNBC cells with or without transfection of hsa_circ_0006466 siRNA. ( ^∗∗^ *p* < 0.01,  ^∗∗∗^ *p* < 0.001.).

**Figure:**
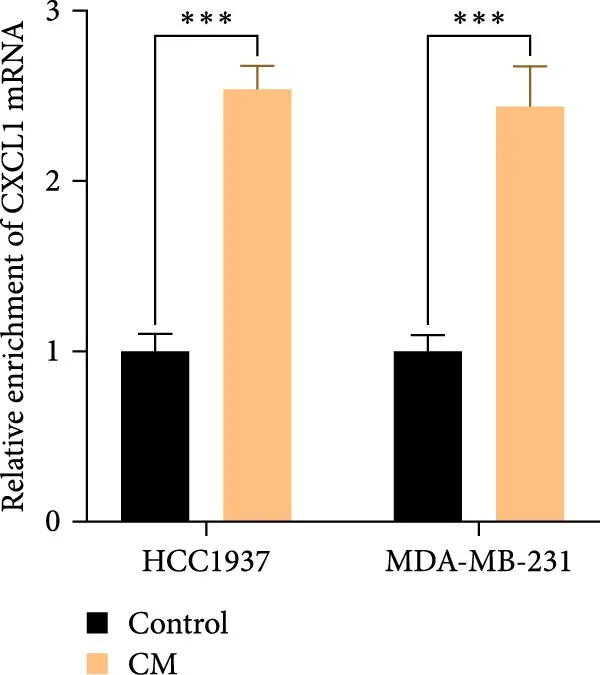
(A)

**Figure:**
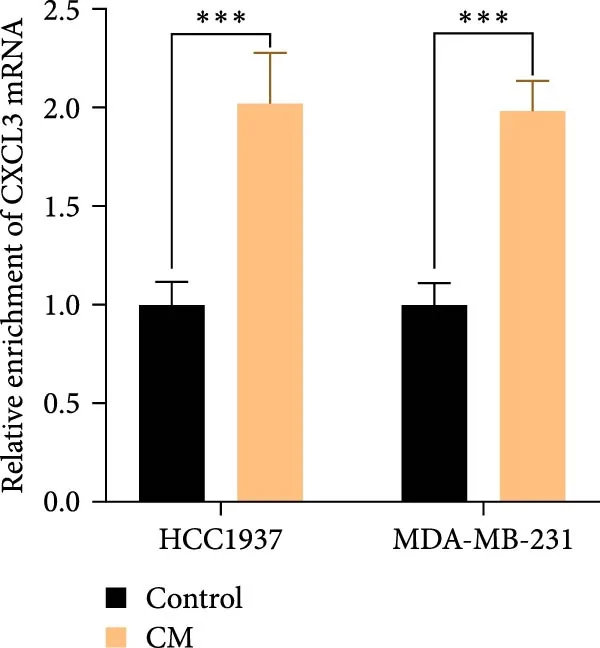
(B)

**Figure:**
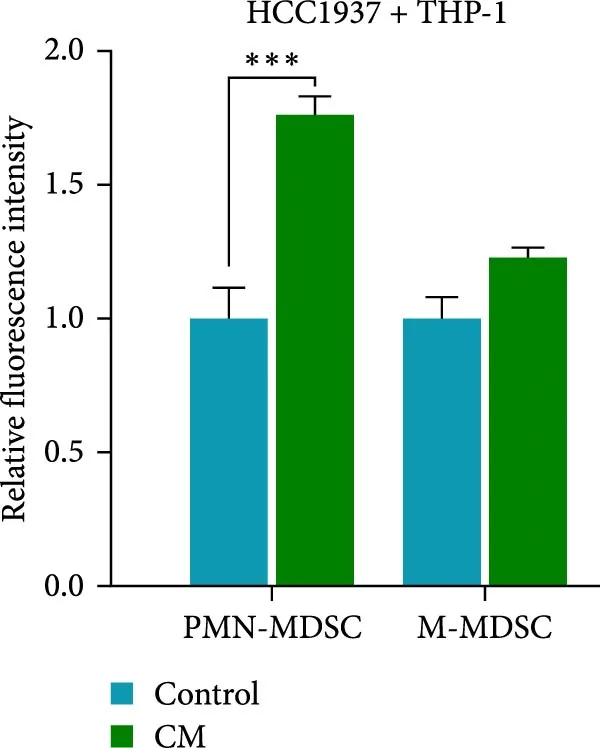
(C)

**Figure:**
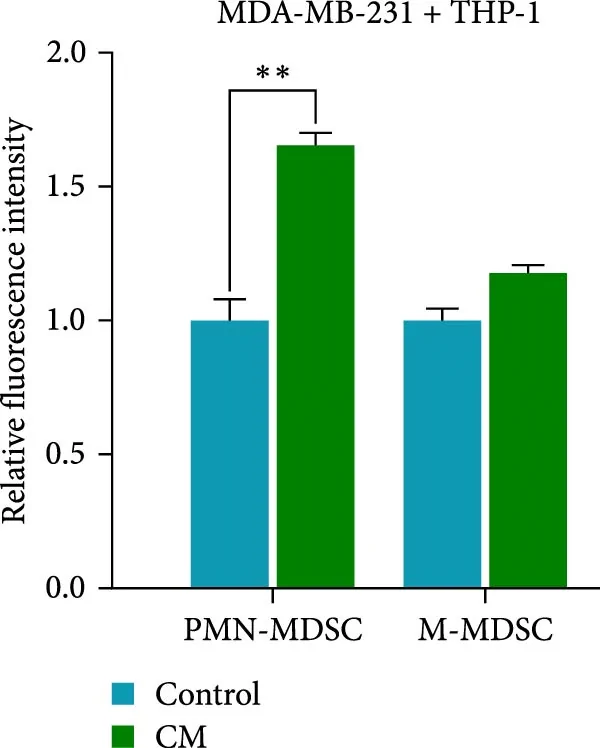
(D)

**Figure:**
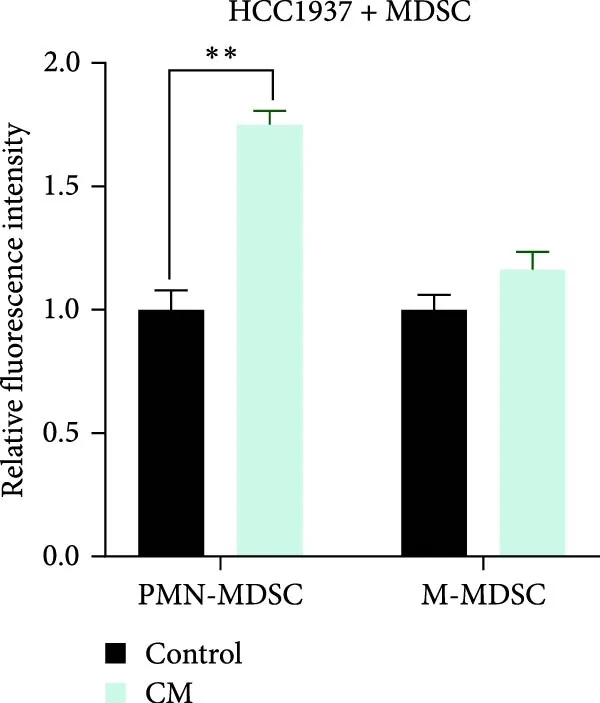
(E)

**Figure:**
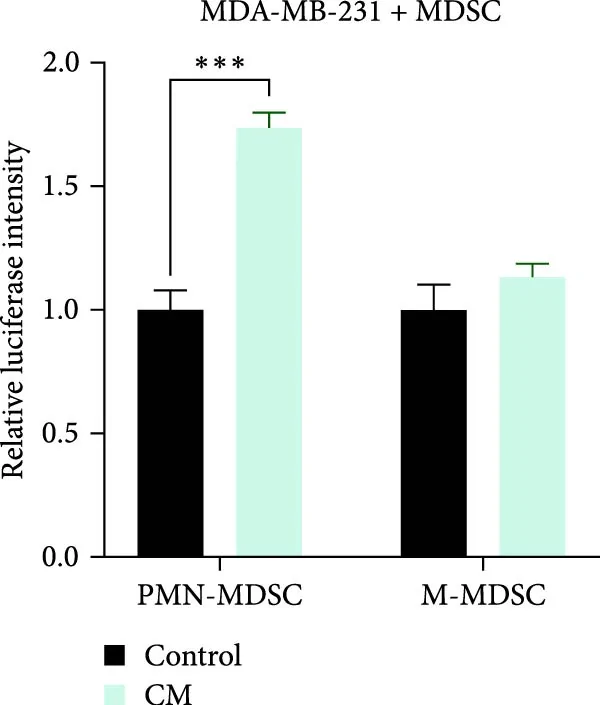
(f)

## 4. Discussion

In recent years, more and more studies have been conducted on the epigenetic mechanism of breast cancer and its potential as a therapeutic target [20, 21]. CircRNA abnormality is a form of epigenetic change [22]. Our preliminary studies identified hsa_circ_0006466 as a novel circRNA upregulated in TNBC tissues and correlated with immunosuppressive TME features. Functional experiments revealed that hsa_circ_0006466 knockdown significantly reduced cell migration/invasion and cell proliferation in TNBC, suggesting its role in anti‐TNBC. The positive correlations of hsa_circ_0006466 expression with MDSC levels imply a role of hsa_circ_0006466 in TNBC immune modulation. Mechanistically, we uncovered a hsa_circ_0006466‐miR‐1286‐*PDGFRA*/*PDGFRB* axis: hsa_circ_0006466 acts as a ceRNA to sponge miR‐1286, thereby derepressing platelet‐derived growth factor receptors A/B (*PDGFRA*/*PDGFRB*). PDGF signaling is known to recruit MDSCs via stromal activation and chemokine secretion (e.g., CXCL1 and CXCL3) [19], but its regulation by circRNA‐miRNA crosstalk in TNBC is unprecedented. These findings position hsa_circ_0006466 as a player in TNBC immune evasion, bridging non‐coding RNA biology with MDSC‐driven immunosuppression.

Hsa_circ_0006466 originates from *UBQLN1*. CircUBQLN1 can be negatively regulated by XBP1s. It can drive osteoarthritis by blocking circUbqln1‐dependent Prodh activation, which dysregulates proline metabolism and collagen synthesis in chondrocytes [23]. CircUBQLN1 has been reported to drive hippocampal neuron proliferation while inhibiting apoptosis and oxidative stress by upregulating *SOX7* via miR‐155 in epilepsy [24]. In our study, we found hsa_circ_0006466 can promote the TNBC cell proliferation. In addition, hsa_circ_0006466 can aggravate TNBC cell migration and invasion. Notably, we found hsa_circ_0006466 upregulation in TNBC patients was positively correlated with MDSC abundance. Cell experiments verified that hsa_circ_0006466 suppression can inhibit the MDSC differentiation from THP‐1, and impede the MDSC chemotaxis. These findings suggest that hsa_circ_0006466 may serve as a critical regulator of MDSC development and migration, potentially modulating immunosuppressive pathways in the TME. CircRERE‐PMN has been identified to be prevalent in MDSCs, supporting its potential as a therapeutic target for lung metastasis of malignant cancer [25]. circ‐E‐cadherin and C‐E‐cad are overexpressed in breast cancer and promote MDSC recruitment and survival, and therefore, targeting it increases the efficacy of immune checkpoint inhibitor therapy [26]. Here, we found targeting hsa_circ_0006466 could represent a novel therapeutic strategy to disrupt MDSC‐mediated immunosuppression in TNBC.

CircRNAs function as miRNA sponges to modulate gene expression, a mechanism well‐documented in cancer biology [27]. Our dual‐luciferase reporter assays confirmed that hsa_circ_0006466 directly binds miR‐1286 via conserved miRNA response elements (MREs), while miR‐1286 targets the 3^′^UTRs of *PDGFRA* and *PDGFRB*. This aligns with the canonical ceRNA model, where circRNA‐miRNA‐mRNA networks drive tumor progression [28]. miR‐1286 is a poorly characterized miRNA with emerging roles in cancer. Reports have revealed its tumor‐suppressive function in gastric cancer [29, 30]. MiR‐1286 can act as a downstream mediator of circHIPK3 in breast cancer, and target *HK2*, thus sensitizing paclitaxel‐resistant breast cancer cells to chemotherapy [31]. Here, we identify *PDGFRA*/*B* as targets of miR‐1286 in TNBC. PDGF ligands (e.g., PDGF‐CC) secreted by TNBC cells activate stromal *PDGFRA*/*B*, inducing chemokine production (CXCL1, CXCL3) that recruits CXCR2^+^ MDSCs [19]. Our data show that hsa_circ_0006466 knockdown reduced CXCL1/3 levels in TNBC cell medium. This corroborates the potential role of hsa_circ_0006466 in shaping immunosuppressive niches. Our work preliminarily demonstrates hsa_circ_0006466‐mediated regulation of PDGF signaling and MDSC biology, expanding the functional repertoire of circRNAs in immune modulation.

## 5. Conclusion

By integrating circRNA biology, miRNA targetomics, and immunology, we unveiled a hsa_circ_0006466/miR‐1286/*PDGFRA*/*B* axis in promoting TNBC progression and immune modulation. The inverse correlation between hsa_circ_0006466 and miR‐1286 supports a ceRNA mechanism, where circRNA sponging of miR‐1286 derepresses *PDGFRA*/*B* to drive MDSC recruitment. Our work advances understanding of circRNA‐mediated immune regulation and potential therapeutic vulnerabilities for TNBC.

## Conflicts of Interest

The authors declare no conflicts of interest.

## Funding

This work was supported by the Qingdao Natural Science Foundation (Grant No. 25‐1‐1‐224‐zyyd‐jch) and the Natural Science Foundation of Shandong Province, China (No. ZR2022MH220).

## Supporting Information

Additional supporting information can be found online in the Supporting Information section.

## Supporting information


**Supporting Information** Figure S1: The intersected dysregulated miRNAs from GSE248460 and circRNA‐targeting miRNAs associated with immune.

## Data Availability

The data that support the findings of this study are available from the corresponding author upon reasonable request.
