# Supplementary material for: Regulation of Cell Function and Myeloid‐Derived Suppressor Cell Chemotaxis by hsa_circ_0006466‐miR‐1286‐PDGFRA/B Axis in Triple Negative Breast Cancer
Source: Anal Cell Pathol (Amst). 2026 Feb 13;2026:2285318. doi: 10.1155/ancp/2285318 (PMC13580802; doi:10.1155/ancp/2285318)
Supplement: Supplementary file 1 [file ANCP-2026-2285318-s001.pdf]

Differential Expressed Genes

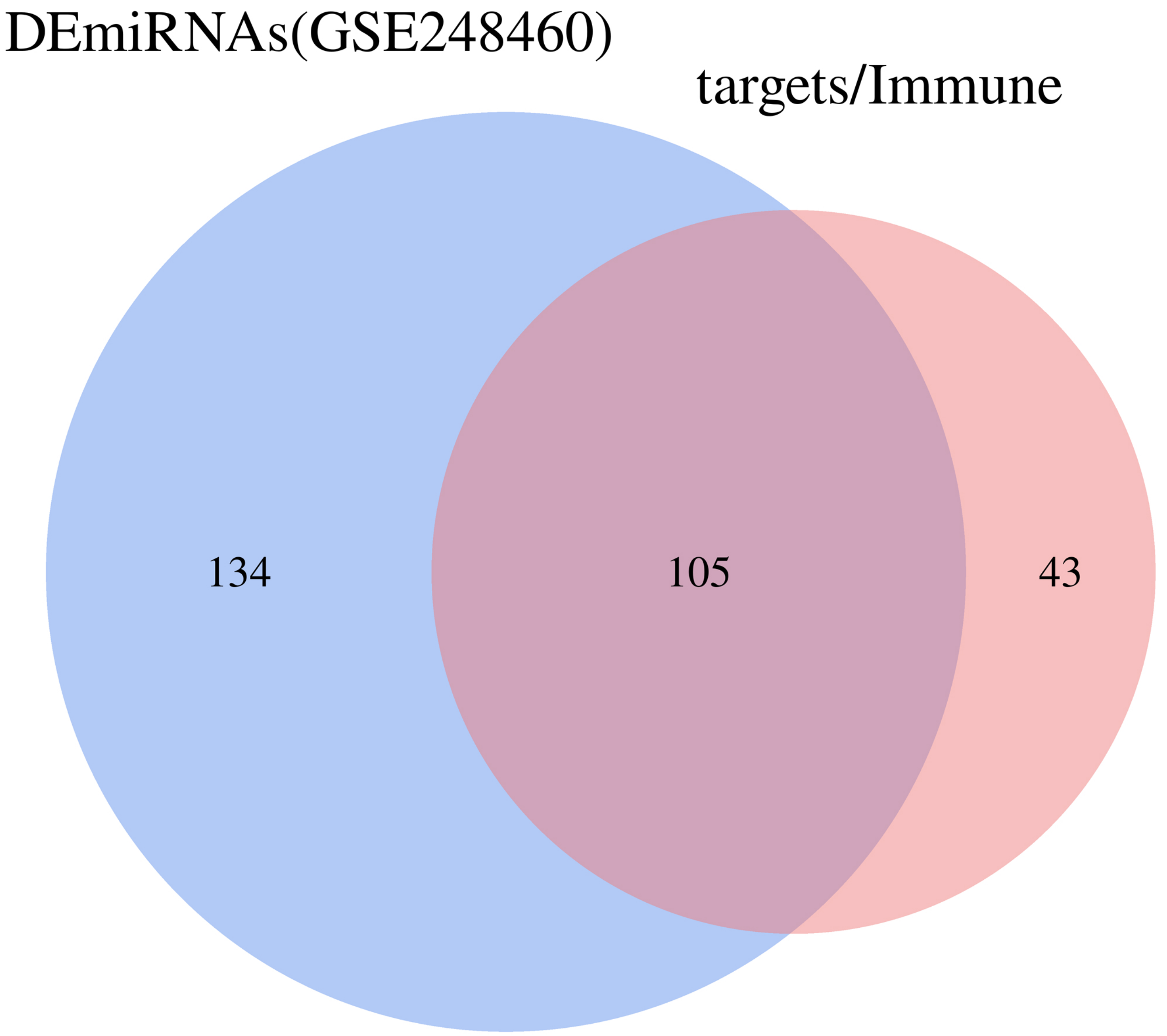

105 common elements in "DEmiRNAs(GSE248460)" and "targets/Immune":

let-7c-3p	miR-143-5p	miR-218-5p	miR-376c-3p	miR-5683
let-7c-5p	miR-145-3p	miR-23a-5p	miR-376c-5p	miR-5701
miR-100-5p	miR-145-5p	miR-2467-5p	miR-378a-5p	miR-574-3p
miR-10b-3p	miR-146b-3p	miR-301a-3p	miR-378i	miR-589-5p
miR-125b-2-3p	miR-148b-5p	miR-3074-5p	miR-3917	miR-592
miR-1260b	miR-151a-5p	miR-30a-3p	miR-422a	miR-659-5p
miR-128-1-5p	miR-152-3p	miR-30e-3p	miR-4326	miR-671-3p
miR-1286	miR-15b-5p	miR-3176	miR-4435	miR-7-5p
miR-1301-3p	miR-17-3p	miR-3184-5p	miR-4443	miR-766-3p
miR-1304-3p	miR-181a-5p	miR-324-3p	miR-4510	miR-767-5p
miR-1307-3p	miR-181b-5p	miR-324-5p	miR-452-3p	miR-7705
miR-1307-5p	miR-181d-5p	miR-330-5p	miR-454-3p	miR-7706
miR-130a-3p	miR-182-5p	miR-331-3p	miR-454-5p	miR-7974
miR-130a-5p	miR-183-5p	miR-337-3p	miR-4703-3p	miR-7977
miR-130b-3p	miR-185-3p	miR-33b-3p	miR-483-5p	miR-877-5p
miR-130b-5p	miR-188-5p	miR-340-3p	miR-484	miR-93-3p
miR-133a-3p	miR-193a-3p	miR-345-5p	miR-486-3p	miR-93-5p
miR-133b	miR-195-3p	miR-3614-5p	miR-486-5p	miR-937-3p
miR-135a-5p	miR-204-5p	miR-3615	miR-497-5p	miR-940
miR-139-3p	miR-21-3p	miR-362-5p	miR-500a-3p	miR-99a-3p
miR-139-5p	miR-211-5p	miR-3690	miR-561-5p	miR-99a-5p
